# Supplementary material for: In Vitro Antileishmanial and Antitrypanosomal Activities of Plicataloside Isolated from the Leaf Latex of Aloe rugosifolia Gilbert & Sebsebe (Asphodelaceae)
Source: Molecules. 2022 Feb 18;27(4):1400. doi: 10.3390/molecules27041400 (PMC8874434; doi:10.3390/molecules27041400)
Supplement: Supplementary file 1 [file molecules-27-01400-s001.zip › molecules-1586235-supplementary.pdf]

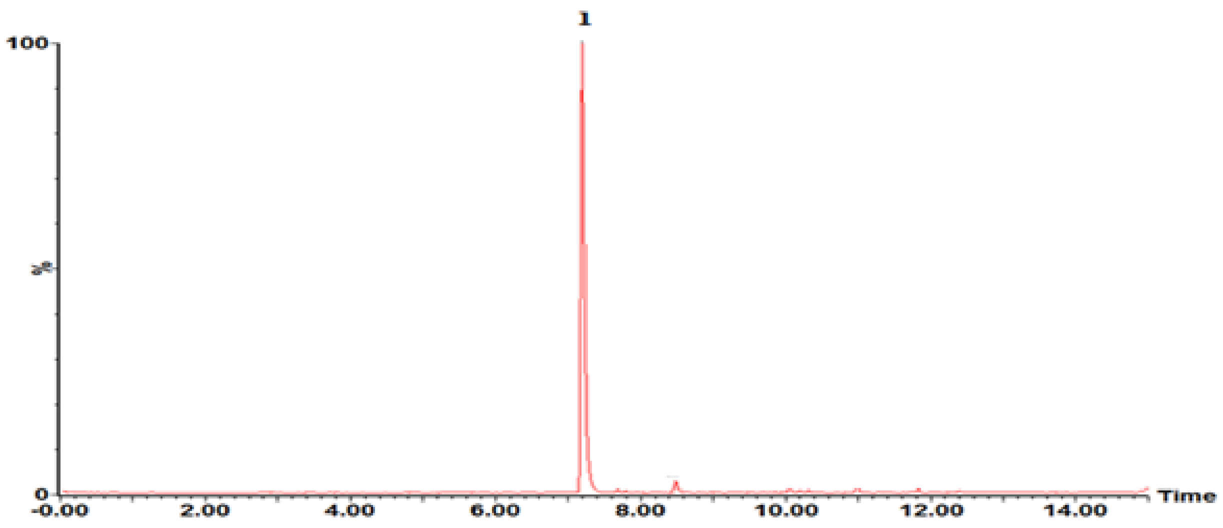

FIGURE S1: HPLC chromatogram of the leaf latex of *Aloe rugosifolia* (plicataloside (1)).

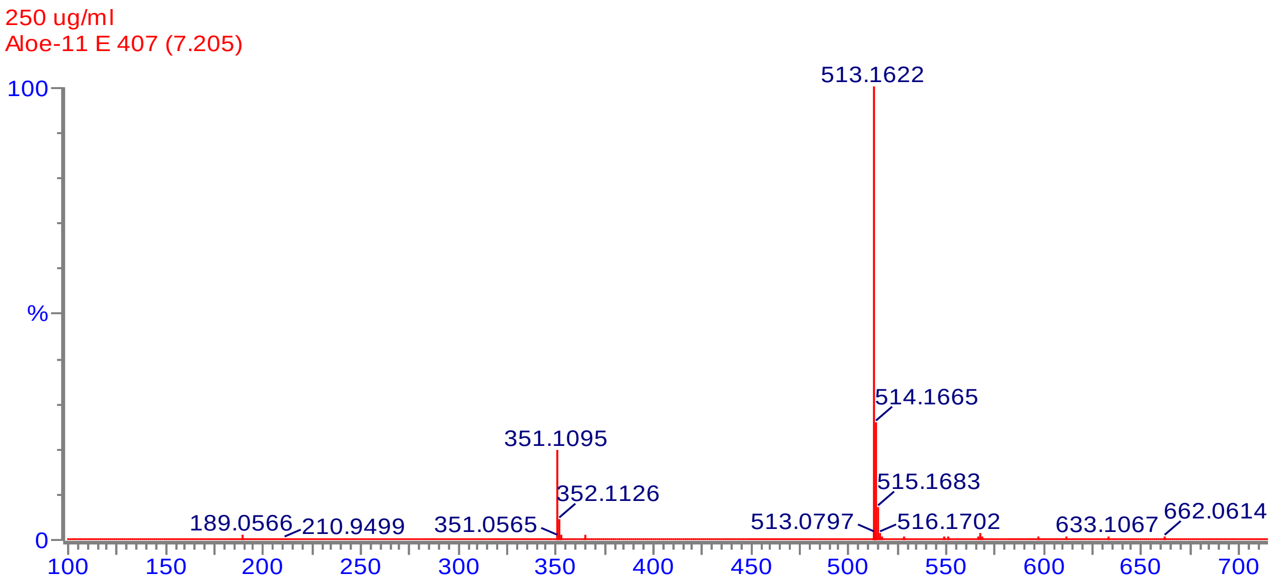

FIGURE S2: Negative-mode HRESI-mass spectrum of plicataloside

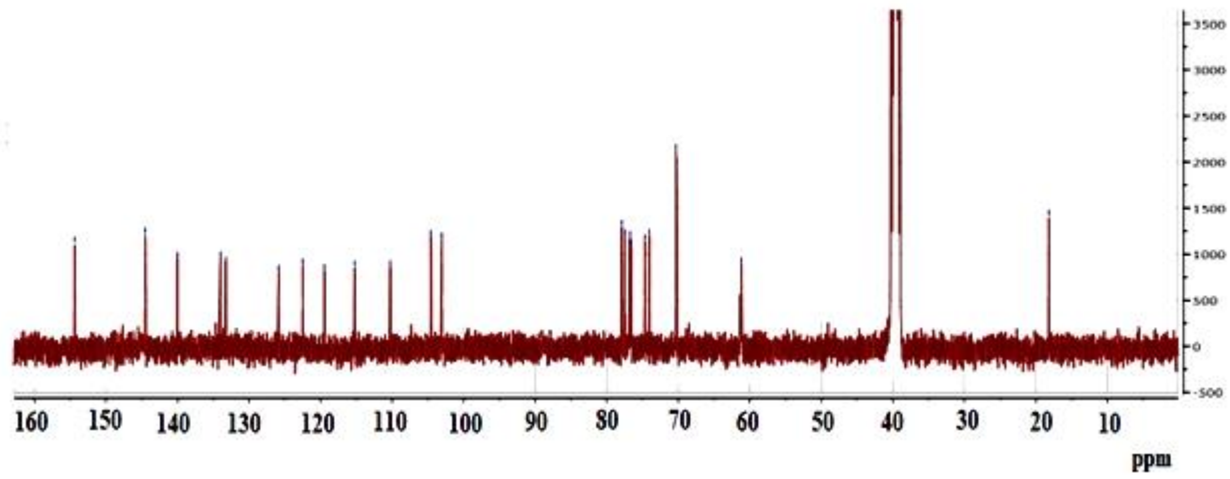

FIGURE S3: ^{13}C-NMR spectrum of plicataloside in DMSO-d_6.

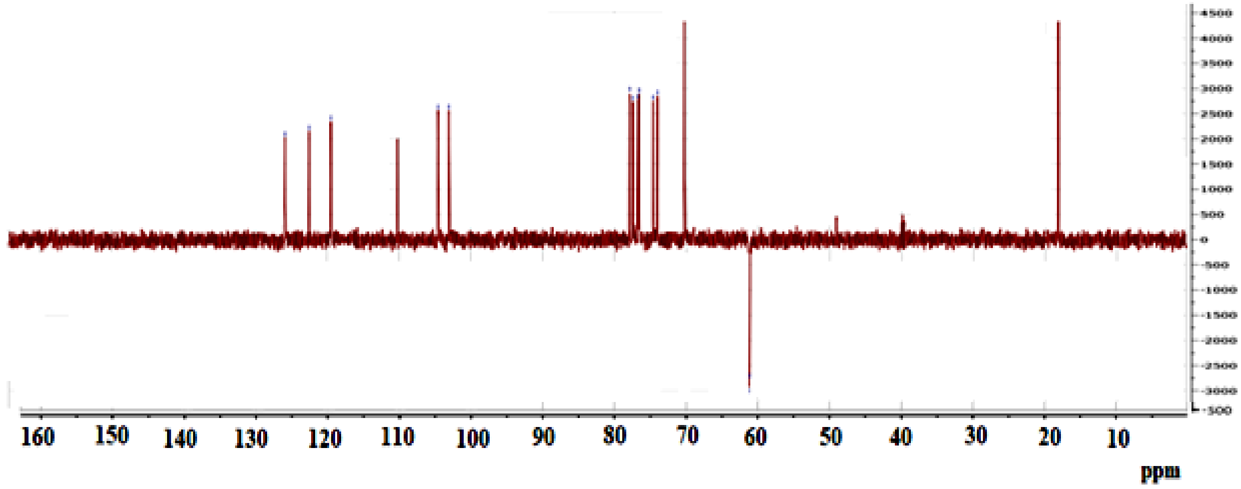

FIGURE S4: DEPT-135 spectrum of plicataloside DMSO-d_6.

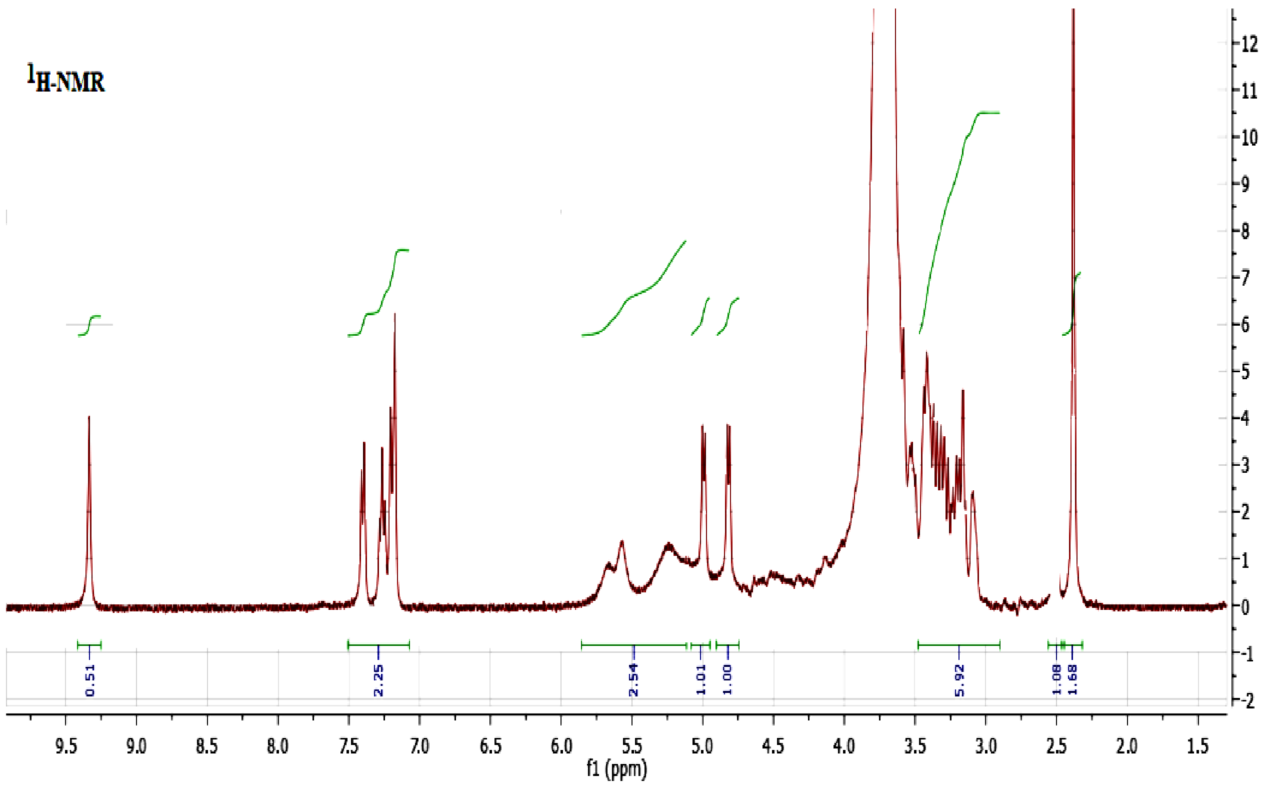

FIGURE S5: ^{1}H-NMR spectrum of plicataloside in DMSO-d_6

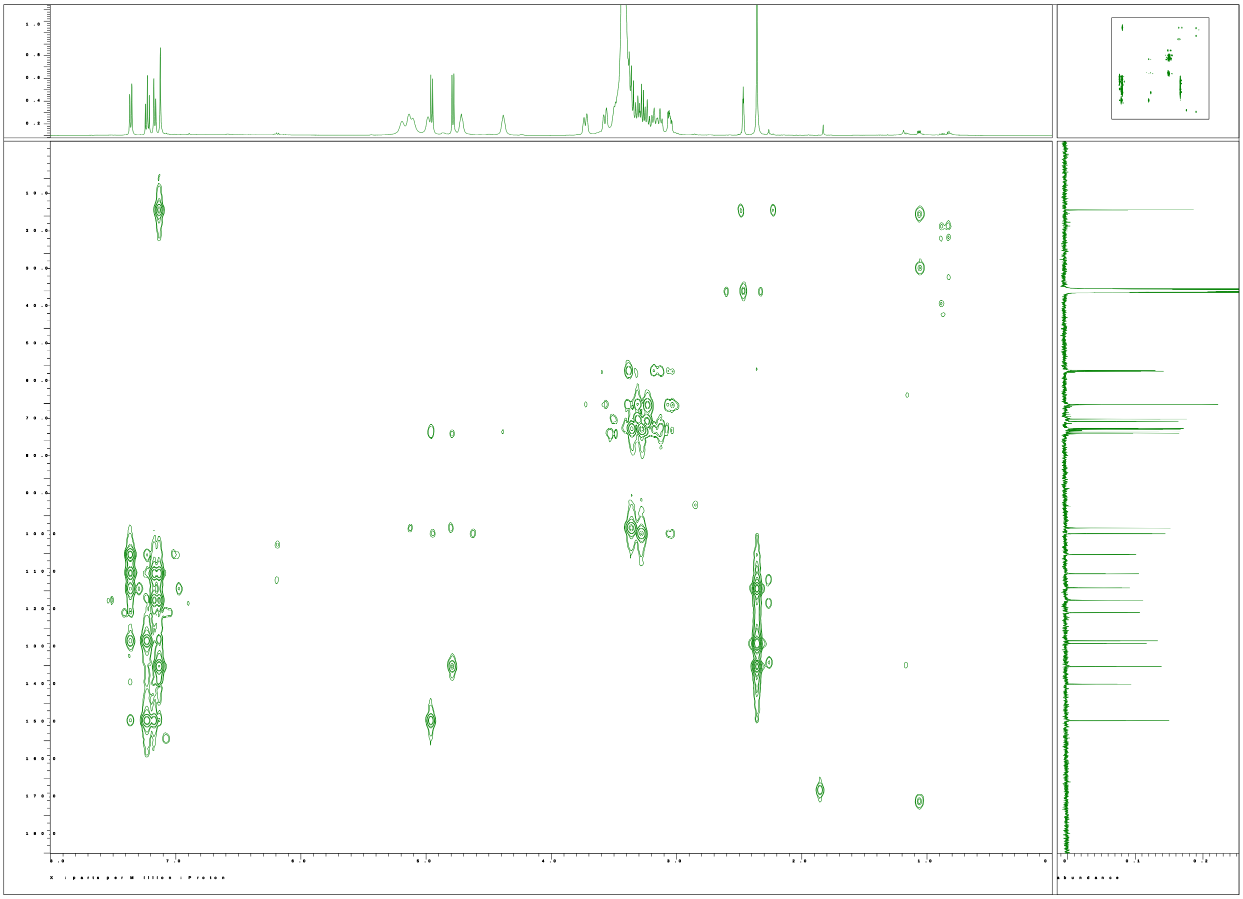

FIGURE S6: HMBC spectrum of plicataloside DMSO-d_6.
